# Comparing tailored implementation strategies to improve intervention fidelity in a school-based obesity prevention program: the IMPROVE hybrid type III trial

**DOI:** 10.1186/s13012-025-01481-0

**Published:** 2025-12-28

**Authors:** Kristi Sidney Annerstedt, Jhon Álvarez Ahlgren, Emma Patterson, Susanne Andermo, Åsa Norman, Sara Raposo, Lydia Kwak, Liselotte Schäfer Elinder

**Affiliations:** 1 Department of Global Public Health, Karolinska Institutet, Stockholm, SE-171 77 Sweden; 2 Department of Risk and Benefit Assessment, Swedish Food Agency, Uppsala, Sweden; 3 Department of Neurobiology, Care Sciences and Society, Karolinska Institutet, Huddinge, SE-141 83 Sweden; 4 Department of Physical Activity and Health, The Swedish School of Sport and Health Sciences, Stockholm, Sweden; 5 Department of Clinical Neuroscience, Karolinska Institutet, Solna, SE-171 65 Sweden; 6 Department of Women’s and Children’s Health, Uppsala University, Uppsala, Sweden; 7 Centre for Epidemiology and Community Medicine (CES), Region Stockholm, Stockholm, SE-104 31 Sweden; 8 Institute of Environmental Medicine, Karolinska Institutet, Stockholm, SE-171 77 Sweden

## Abstract

**Background:**

In Sweden, childhood overweight and obesity rates have risen significantly over the last decades, necessitating scalable interventions. The evidence-based Healthy School Start (HSS) program integrates school and family components to promote healthy habits and prevent overweight and obesity among children. The IMPROVE trial aimed to compare the effect of two tailored implementation strategy bundles (Basic and Enhanced) on fidelity to the HSS program.

**Methods:**

A hybrid type III cluster-randomized trial with two parallel arms was conducted in 45 schools (cluster) in three municipalities in Stockholm Sweden from August 2021 to June 2024. The program was implemented in two consecutive cohorts over two academic school years. Fidelity was measured with an adherence score (0–4) and parent’s responsiveness (1–5) to the four intervention components (health brochure, motivational interviewing health talk, classroom module and type 2 diabetes risk test). Data were analyzed using mixed-effects linear and logistic regression models.

**Key findings:**

A total of 946 parents and 655 children participated. Overall fidelity, assessed as an adherence score, was around 75%, with most components implemented as expected. The adherence score in the Basic bundle showed no significant difference compared to the Enhanced implementation strategy bundle (β = 0.01, *p* = 0.95, 95% CI: –0.24, 0.25). Two of four Enhanced implementation strategies, educational outreach visits and networking between school and primary health care, did not happen mainly due to lack of interest and time among personnel. Parents born within the Nordic countries had twice the odds (*p* < 0.001, 95% CI: 1.14–3.43) of completing the motivational interviewing health talk compared to those born outside the Nordics.

**Discussion:**

Enhancing the Basic implementation bundle with additional strategies did not consistently improve adherence or responsiveness. However, improvements observed over time underscore the importance of targeted support during the initial implementation year. Additional motivational actions might be needed in schools with a high proportion of children whose parents are born outside the Nordic region. These findings highlight the complex interplay between context and implementation success, emphasizing the need to adapt strategies over time to optimize their effectiveness rather than merely adding more. Moreover, the essentially null findings also point to broader methodological challenges in implementation science, particularly how to prioritize among determinants, strategy selection and tailoring.

**Trial registration:**

ClinicalTrials.gov, Unique Protocol ID: NCT04984421. Registered July 30, 2021, https://register.clinicaltrials.gov/.

**Supplementary Information:**

The online version contains supplementary material available at 10.1186/s13012-025-01481-0.

Contribution to literature

- This study contributes to the limited evidence on implementation strategies for complex health promotion school-based interventions comparing a more intensive, tailored strategy bundle to improve fidelity to a basic approach.
- It is among the few hybrid type 3 implementation studies to assess fidelity not only to the intervention itself but also to the implementation strategies in two consecutive cohorts.
- The findings highlight the importance of comprehensive and multi-perspective fidelity assessments by both deliverers and recipients.
- The study underscores the need for adaptive implementation strategies in complex and multi-layered systems such as schools.

## Background

Unhealthy diets and physical inactivity are among the leading risk factors contributing to the global burden of disease, including in Sweden, where non-communicable diseases such as obesity, and type 2 diabetes (T2D) continue to rise [1]. In Sweden, the prevalence of childhood overweight and obesity has doubled over the past three decades, with children in socioeconomically disadvantaged areas being 2–3 times more likely to be affected by obesity than those in more affluent areas [2]. Likewise, children of non-Nordic born parents have more overweight and obesity than children from Nordic-born parents, partly due to a higher intake of unhealthy foods and drinks [3]. These disparities are largely driven by social and environmental factors (e.g., socioeconomic status, cultural norms, food marketing, neighborhood walkability, and access to healthy foods) that shape dietary and physical activity behaviors [2]. Addressing behavioral determinants requires evidence-based, scalable interventions that can effectively support children and families in adopting healthier lifestyles.

In response, the Swedish national guidelines recommend family support programs to help manage unhealthy diet and physical inactivity in children [4]. Schools are widely recognized as ideal settings for delivering health promotion programs, particularly for children aged 6–12 [5, 6]. Moreover, interventions that actively engage parents tend to be more effective, as parents play a critical role in shaping the home food environment, physical activity opportunities, and social norms around health [7]. However, no evidence-based program has been available in Sweden that systematically integrates both school and family components.

To address this gap, our research group started to develop the Healthy School Start (HSS) program in 2010, grounded in Social Cognitive Theory (SCT) [8]. SCT explains behavior as the reciprocal interaction between personal, behavioral, and environmental factors. A central construct is self-efficacy, belief in one’s ability to perform a specific action. HSS targeted parental self-efficacy (e.g., serving vegetables at each meal), alongside other relevant SCT constructs such as observational learning, behavioral capability, outcome expectations, and self-regulation. The program is universal, delivered to all children regardless of weight status in the grade, and aimed at both health promotion and obesity prevention. Three cluster-randomized trials, including almost 1000 families mainly in disadvantaged areas in and around the greater Stockholm region have been conducted, showing similar beneficial effects [8–10]. We found significant effects on increased consumption of fruit and vegetables, and decreased consumption of unhealthy foods and drinks [8, 10] and in the second trial also a significant decrease in body mass index (BMI) z-score in children with obesity at baseline compared to control [8, 9]. The beneficial outcomes on diet were largely confirmed in the third trial [10]. When pooling the data from all three trials, we were able to show that the BMI z-score among children with obesity had decreased significantly, approaching the effects seen in obesity management trials [11].

Many interventions fail to sustain impact over time due to challenges in scaling up and maintaining high-fidelity implementation [12]. A range of multilevel factors influence implementation success [13]. While high fidelity, scalability, and long-term sustainability are essential, complex interventions often fail because context-specific barriers are overlooked, and implementation support is insufficient leading to low prioritization by stakeholders. The Consolidated Framework for Implementation Research (CFIR) identifies factors across domains, such as stakeholder perceptions, organizational readiness, and client prioritization [14]. To improve implementation and ultimately promote student health, strategies must be tailored to address these specific factors [15].

A review highlighted determinants for the successful implementation of obesity prevention interventions for children with low socioeconomic status included factors such as lack of time and communication, however factors in the *outer setting* i.e., external influences that affect implementation efforts within an organization were often not explored [16]. Tailoring implementation strategies to the most critical contextual determinants is essential for improving fidelity, which encompasses adherence, dose, quality of delivery, participant responsiveness, enactment, and program differentiation [17].

The IMPROVE trial (IMplementation and evaluation of the school-based family support PRogram a Healthy School Start to promote child health and prevent OVErweight and obesity) described in this paper represents the next step towards sustainability of the HSS program. Key factors for scaling up school-based interventions are many and include systematic use of evidence, establishment of monitoring and evaluation systems, calculation of costs, active engagement of the target community through a participatory approach, tailoring to the local context, infrastructure to support implementation, strong leadership and champions, political will, a well-defined scale-up strategy and strong advocacy [18]. Thus, a combination of strategies will most likely be required to achieve high fidelity and clinical effectiveness. With this in mind, the aim of the IMPROVE study [19] was to compare two bundles of tailored implementation strategies (Basic and Enhanced) to assess their impact on fidelity to the four intervention components in the HSS program. The hypothesis was that the use of additional tailored implementation strategies in the Enhanced bundle with a focus on facilitation, monitoring, and feedback would lead to better fidelity to the HSS intervention components compared to the Basic bundle.

## Methods

### Trial design

The IMPROVE study was a hybrid type III cluster-randomized trial [20] with two parallel arms testing two bundles of implementation strategies—Basic (group 1) and Enhanced (group 2). The primary outcome was fidelity to the intervention components. The study was conducted between 2021 and 2024 in three municipalities with somewhat higher health needs in the greater Stockholm region. Two municipalities started the trial in 2021 (M1 and M2) and the third (M3) in 2022. School principals were informed by the local educational department about the municipality’s intention to implement the program in all public schools. If a school had special reasons or circumstances such as staff shortages, the principal had the possibility to decline participation.

Within each municipality, schools were randomly assigned to receive either the Basic or an Enhanced Bundle of implementation strategies. To minimize “contamination” between groups, we applied contingent allocation in cases where a school nurse worked across more than one school. In these instances (eight schools across four nurses), all schools served by the same nurse were assigned to the same study arm. Randomization was conducted by an independent statistician who was blinded to the schools and not involved in the project. While it was not possible to blind school staff or research team members to allocation, parents and children were not informed about whether their school was assigned to the Basic or Enhanced Bundle of implementation strategies. Importantly, all parents and children received the same Healthy School Start program regardless of trial arm.

The study is reported according to the Consolidated Standards of Reporting Trials (CONSORT Cluster) Statement [21] (Additional file 1) and the Standards for Reporting Implementation Studies (StaRI) guidelines [22] (Additional file 2).

### Study setting and participants

In the fall of 2020, eight municipalities out of 26 in the Stockholm Region, Sweden were contacted to gauge their willingness to participate in the IMPROVE study. These municipalities had high rates of overweight and obesity among 4-year-old children [19]. Key personnel in three municipalities (M1-M3), including central administrators and school healthcare coordinators responded positively and received an online introductory meeting explaining the intervention and the study. After discussions with their education administrators, M1 and M2 agreed to participate immediately and M3 agreed a year later which was after publication of the study protocol, mainly due to the Covid-19 pandemic. Table 1 outlines relevant information about M1-M3.

**Table 1:** Description of the population, schools and health in involved municipalities (M)

	**Sweden**	**M1**	**M2**	**M3**
**Population characteristics**				
Number of inhabitants^1^	10 400 000	114 000	50 000	101 000
Median income per year (SEK)^1^	460 000	333 000	307 000	289 000
Proportion unemployed (%)^1^	7.4	7.4	9.1	11.4
Proportion with ≥ 3 years university education 25–64 years (%)^1^	30.3	32.4	21.9	22.3
Proportion of foreign-born inhabitants (year 2021)^1^ (%)	20.0	30.8	36.2	42.4
**School characteristics**				
Proportion of certified teachers (2021) (%)^2^	71.0	74.5	59.9	66.7
School rank out of 290 municipalities (combination of teachers’ salary, teachers’ health and teacher density)^3^	-	214	219	232
Density of students per teacher (rank out of 290 municipalities)^3^	-	288	251	200
**Health Indicators**				
Overweight (not obesity) 4-year olds born 2017 (%)^4^	10.9	9.4	11.7	11.4
Obesity 4-year olds born 2017 (%)^4^ or 2016^5^	2.9	4.1	5.3	5.1
Overweight and obesity in individuals ≥ 16 years (self-reported) (%)^6,7^	51.0	51.3	53.5	56.1
Diabetes in individuals ≥ 16 years (self-reported) (%)^6,8^	4.4—5	3.3	6	6.1

^1^Central Bureau of Statistics – Statistiska Centralbyrån SCB. Kommuner i siffror [Internet]. Stockholm: SCB; 2021/2022

^2^Skolkollen.se 2023

^3^Swedish Teacher’s Union – Lärarförbundet. Bästa skolkommun—resultat och historik [Internet]. Stockholm: Lärarförbundet; 2021. The ranking is based on quality with determinants such as resources, number of children in preschool class, educated and healthy teachers, teachers per student, and student gradings

^4^Barnhälsovårdens Årsrapport 2022 (Yearly report Child Health Care), Region Stockholm

^5^Miregård et al. Acta Paediatrica 2023

^6^Folkhälsokollen.se, Region Stockholm

^7^Public Health Agency of Sweden

^8^Diabetics Association in Sweden

A total of 45 schools enrolled. Within these schools, all personnel relevant to the HSS program (i.e., principals and vice principals, school nurses, and teachers, see below) were invited to join the study. Parents or guardians (henceforth referred to as parents) of children entering preschool class (ages 5–6) in M1 and M2 during the academic years 2021/22 and 2022/23 were also eligible for the study. In M3 the children started the program in grade 1 (ages 6–7) during the 2022/23 and 2023/24 academic years. The HSS program was introduced to parents as part of routine school activities early in the fall term, and information sheets and consent forms were distributed to all families, emphasizing that participation in the study was voluntary. Participants who consented completed surveys (online or on paper if requested) at three timepoints: baseline (fall, before the program started), post-intervention (spring, after program completion), and one year later. These surveys collected data on process and outcome measures. Only data from the first two timepoints were used in this study.

### The Healthy School Start (HSS) program

The HSS is a universal program targeting all preschool class or first grade children over the course of one academic year. It comprises four key components including a parent brochure with health information in five languages, motivational interviewing (MI) health talks with parents performed by the school nurse directly after the routine health visit, nine classroom activities with home assignments performed by teachers, and a type 2 diabetes (T2D) self-assessment risk test for parents. More details of the intervention can be found in the HSS study protocol [23].

#### Implementation strategies

The IMPROVE study builds on three previous hybrid type I cluster-RCTs [8–10, 23]. Based on this foundation, we employed several pre-implementation strategies as described in detail in the study protocol [19], including local needs assessments, enhancing implementer buy-in (e.g., free MI training for school nurses), obtaining formal commitments, and assessing readiness.

The program theory for the IMPROVE study, where recruitment was done at the municipality level, was developed through a targeted three-step consultation process [24]. First, school personnel responsible for preschool class and grade 1 in M1 and M2 participated in online workshops where barriers and facilitators to implementation were identified and coded into 28 determinants [19]. Second, the research team organized these determinants using the Consolidated Framework for Implementation Research (CFIR) [25]. The team then systematically reviewed the SISTER taxonomy [26], which provides a standardized classification of implementation strategies for school settings, to identify strategies that addressed all determinants and in turn, developed two implementation strategy bundles: Basic (group 1) and Enhanced (group 2). The Basic bundle included strategies considered essential for implementation at school level, such as consensus-building discussions, formation of implementation teams, peer-assisted learning, assessing the needs for changes to the school environment, and preparation of families and students (Table 2). The Enhanced bundle consisted of four additional strategies providing external support by facilitating more direct contact between the research team and school personnel to strengthen implementation and lead to higher fidelity. These strategies were outreach visits, promoting network weaving, ongoing consultation (coaching), and providing feedback. Lastly, in a second workshop, the same school personnel were invited to refine the proposed strategies and provide input on their form and function. The scientific advisory panel was then consulted, resulting in minor adjustments as documented in the study protocol [19].

**Table 2:** Description of implementation strategies and necessary adaptations made during the implementation phase

	**Implementation strategies (SISTER taxonomy number)**	Maximum for checklist score^*^	**Description of the strategy**	**Adaption to implementation strategy**	**Actors**	**Target**
**Basic Bundle (1–6)**	1. Conduct local consensus discussions (23)	2	Introduction of the HSS to all school personnel with the help of material to create commitment and reach consensus	None	School principal	School personnel
	2. Distribute educational materials (42)	1	Order educational material at the beginning of the new school year (implementation manual, teachers manual, workbook for children, posters etc.) and distribute it	In two municipalities, the research team directly facilitated the ordering of materials with the schools. In the third municipality, a central resource liaised with the schools and placed one order with the research team. All materials were distributed	School health team	School personnel
	3. Organize school personnel implementation team meetings (32)	3	Form a health team including a teacher representative and appoint a coordinator to follow up and reflect on how to divide the practical work with the HSS, the implementation process, knowledge exchange, and how to support one another’s learning	None	School principal and health team	School health team
	4. Peer-assisted learning (13)	9	Watch the introductory video to the classroom component, discuss a plan for implementation, how to engage parents in the home assignments	None	Teachers	Teachers
			Exchange of knowledge and experience in MI	None	School nurses	School nurses
			Listen to educational lectures posted on the HSS website at least once every school year whenever it fits with the schedule	None	School health team	School personnel
			Internal and external meetings for inspiration, knowledge exchange and how to organize the work	None	School health team	School personnel
	5. Change/alter environment (54)	N/A	Discussion regarding the possibility to make changes to support healthy lifestyle within and around the school	None	School health team	Community, school and after-school care
	6. Prepare families and students to be active participants (55)	3	Announcement that the school is a health promoting school	None	School principal	Parents
			Introduction of the HSS at the first meeting with new parents through a film, giving the rational for the intervention, in basic Swedish and texted in other common languages	None	School personnel	Parents
			Information sent out in the newsletter to families of the start of the HSS. Encouragement to consult the HSS website for further information and material	None	School personnel	Parents
**Enhanced Bundle (1–10)**	7. Conduct educational outreach visits (38)	1	Yearly presentations to schools on topics in public health of their choice and relevant to the intervention	This was offered to each school in group 2 during the coaching sessions (one in the fall term and one in the spring term), but none of the schools identified a need for additional information	Research team	School personnel
	8. Promote network weaving (33)	1	Send out yearly information letter to primary health care centers about the HSS program and the IMPROVE study. Encourage yearly meetings to establish social networks, promote information sharing, collaborative problem-solving and shared goals regarding family health	None, but no meetings took place	Research team	School health care and primary care
	9. Provide ongoing consultation/coaching (44)	2	Yearly audit and feedback through a written report on the fidelity score and performance of implementation strategies with coaching how to improve. E-mail sent four times per year to the school health team coordinator to offer assistance and help with problem solving	Schools received bi-yearly feedback through an onlinemeeting with a PowerPoint presentation from the research team. Improvements were then instead offered verbally in the coaching meetings	Research team	School personnel
	10. Obtain and use student and family feedback (8)	1	Yearly feedback on parents’ attitude and perception of the program	Parent feedback was shared with the school during the first coaching meeting in the second year, as it was collected after the completion of the first year. Schools that only implemented HSS in the first year did not receive this feedback	Research team	School personnel

^*^The number of points given for each strategy corresponds to the number of questions asked to school personnel

**Fig. 1:**
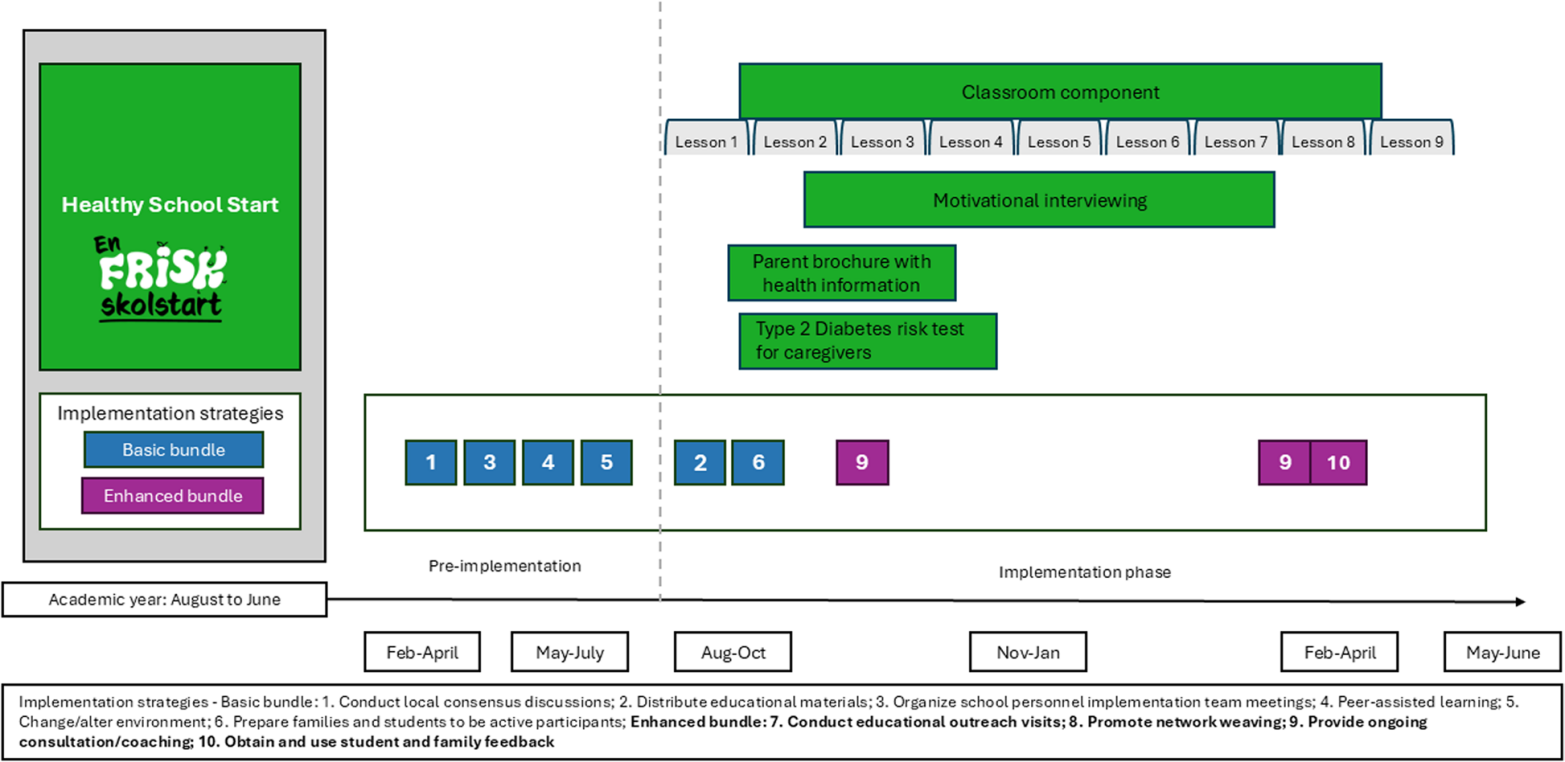
Pictorial illustration of the implementation of the Healthy School Start program and the two bundles of implementation strategies (Basic and Enhanced)

Changes were made to the form (but not function) of some implementation strategies in both the Basic and Enhanced Bundles about three months after starting the trial as described in Table 2. The implementation of the Healthy School Start program and the two bundles is described in Fig. 1. Other minor deviations from the study protocol are described in Additional file 3. Both the intervention components and the implementation strategies are described in a manual (available in Swedish from the website https://ki.se/gph/forskning/en-frisk-skolstart) provided to all involved school personnel.

Pre-implementation, all school personnel (principals, school nurses and teachers) received separate one hour introductory on-line training on how to perform the intervention components and implementation strategies in practice, access to a central website to assist implementation with extra material for the teachers and the opportunity to ask questions to the research team.

### Data and data collection

#### Primary outcome: adherence to the four components of the Healthy School Start program

Fidelity to the four components of the HSS intervention, the primary outcome, was assessed at 12 and 24 months using eight indicators based on adherence and participant responsiveness. The primary outcome was operationalized as a total adherence score ranging from 0 to 4, based on completion of each intervention component (Table 3). Cohort 1 (adherence measured at 12 months) corresponded to the end of the first academic year, while cohort 2 (adherence measured at 24 months) represented the second year of implementation. Data on adherence to the *health information brochure* (component 1) and the *type 2 diabetes risk test* (component 4) were collected via online questionnaires sent to parents, with an option to respond on paper. Distribution and data collection were managed through the secure REDCap platform hosted at Karolinska Institutet [27]. Adherence to the *motivational interviewing (MI) health talk* (component 2) was reported by school nurses in M1 and M2. In M3 information was obtained centrally from the municipality school journal system. Completion of *classroom activities* (component 3) was confirmed by teachers during annual meetings with researchers. Each component was scored as 0 (not adherent) or 1 (adherent). Missing values were assumed to indicate non-completion and were set to zero.

**Table 3:** Intervention components and adherence and responsiveness to the Healthy School Start program

Component	Adherence (%) (yes = 1 no = 0)	Parents’ responsiveness (scale 1–5)
1. **Parent brochure with health information**	Parents reading the brochure (P)	Parents’ appreciation of the brochure (P)
2. **Motivational Interview (MI) health talk with parents**	Parents receiving MI (N)	Parents’ appreciation of MI (P)
3. **Classroom activities with home assignments**	Teachers providing the classroom component (T)	Parent’s appreciation of the home assignments (P)
4. **Type 2 Diabetes (T2D) Risk test for parents**	Parents doing the test (P)	Parents’ appreciation of the test (P)
**Total score**	Total score (0–4)	Total score not calculated

Informants: *P* parent, *N* nurse, *T* Teacher

#### Participants’ responsiveness and sociodemographic characteristics

Participant ‘responsiveness’ measured how parents responded to and were engaged in the intervention components collected through the same online questionnaires described above. It was measured on a scale from 1 to 5 (no appreciation to full appreciation) for each component.

At baseline, parents also answered questions about their sex, educational level, and country of birth. Parents’ education was classified into four categories: elementary/middle school, high school, post-secondary education, and college/university. Country of birth was categorized as either "Born in a Nordic country" (Denmark, Finland, Iceland, Norway, and Sweden) or "Born outside of the Nordic countries" (all other countries).

If the participants did not fill in the questionnaire, three reminders were sent automatically via email three weeks apart. If a phone number was provided during the informed consent process, an additional three text message reminders were sent and followed up by a phone call reminder if necessary.

#### Fidelity to implementation strategies

The secondary outcome, fidelity to implementation strategies (Table 2), was measured at the school level using a 29-item checklist (Additional file 4) developed by the research team. The checklist was based on the SISTER taxonomy [26] and designed to capture how school personnel applied the implementation strategies described in the study protocol (Tables 2 and 3). It included 22 closed ended (scored dichotomously, 0 = no, 1 = yes) and seven open-ended questions. The maximum score for schools receiving the Basic Bundle was 18 points with schools in the Enhanced Bundle able to receive up to five additional points.

Two data collection rounds were conducted: One in-person visit at month 12 and one digital meeting at month 24. Each one-hour session was facilitated by two to three researchers and involved school personnel responsible for implementation. In total, the checklist was administered to 242 individuals across 73 school visits (44 at month 12 and 29 at month 24), covering 44 schools. Four sessions could not be scheduled due to school personnel’s lack of time. The findings from the checklist meeting were used to provide targeted feedback during the coaching meetings with the schools receiving the Enhanced Bundle in the subsequent year.

One implementation strategy, *“Change/alter environment” (#5),* was removed from the checklist, as most schools considered it redundant. Swedish schools already provide free, nutritious meals and emphasize physical activity, reducing the need for environmental changes.

#### Assessment of readiness

Readiness to implement the program was assessed with a modified version of the Organizational Readiness for Implementing Change (ORIC) tool developed by Shea et al. including 13items each answered on a 5-point Likert scale [28]. Data was collected from school personnel from all 45 participating schools as described previously [29]. The median score was calculated and then schools were divided into low or high readiness.

### Sample size

The primary outcome was fidelity to intervention components assessed through an adherence score and a difference in the log odds of the groups of 0.3, 0.4 or 0.5 (maximum score 4) corresponding to odds ratios of 1.35, 1.5 and 1.65, respectively was assumed in the original sample size calculation. The appropriate sample size for our study was 400 in each group with a significance level (alpha) set at 0.05 and a desired power of 0.80. The study had 45 clusters (schools) with a mean cluster size of 21 participants and standard deviation of 18.

### Statistical analysis

The primary outcome adherence score was assessed at the individual level at month 12 (cohort 1) and month 24 (cohort 2) and described using descriptive statistics. To evaluate the effect of the two bundles of implementation strategies on adherence to the HSS program, we fitted a mixed-effects linear regression model. A random intercept for school was included to account for clustering, and parents’ region of birth and cohort number were included as covariates (Model 1) [19]. The other primary outcome responsiveness score could not be calculated based on all four components because many parents did not answer the question on responsiveness to the MI health talk. We understood that the parents were not always able to distinguish between the regular health talk that came first as part of routine care and the motivational interviewing. If they answered “no” to having received MI, the skip logic in REDCap prevented them from receiving the follow-up question about their opinion of the MI (responsiveness), even though the nurse had documented that the MI had been delivered. Therefore, only the adherence score was used in the statistical analysis of fidelity to the intervention. Cohen’s d was calculated to estimate the standardized mean difference between the intervention and control groups.

To further explore the individual components of the adherence score (i.e., completion of the health brochure, motivational interviewing (MI) session, and type 2 diabetes (T2D) risk test), separate mixed-effects logistic regression models were estimated for each component, adjusting for the same covariates as in Model 1, now referred to as Models 2–4. To explore the impact of the implementation bundles on the responsiveness score (1–5), we fitted a mixed-effects linear regression model for each of the four intervention components.

To explore potential effect modification, subgroup analyses were conducted by including interaction terms between group and (1) school size (categorized as high vs. low based on the median number of students), (2) school personnel readiness for implementation (high vs. low), (3) parental education level (university vs. non-university), (4) parents’ region of birth (Nordic vs. non-Nordic), and (5) cohort (month 12 vs month 24). To account for multiple comparisons, we considered interaction effects statistically significant at *p* ≤ 0.01. Results are reported as regression coefficients (β) for continuous outcomes and odds ratios (ORs) for binary outcomes, each with corresponding *p*-values and 95% confidence intervals. Statistical significance was defined as *p* < 0.05.

The mean score and proportion of maximum (%) score for implementation strategies were calculated for schools assigned to the Basic Bundle (five strategies) and to the Enhanced Bundle (nine strategies).

## Results

**Fig. 2:**
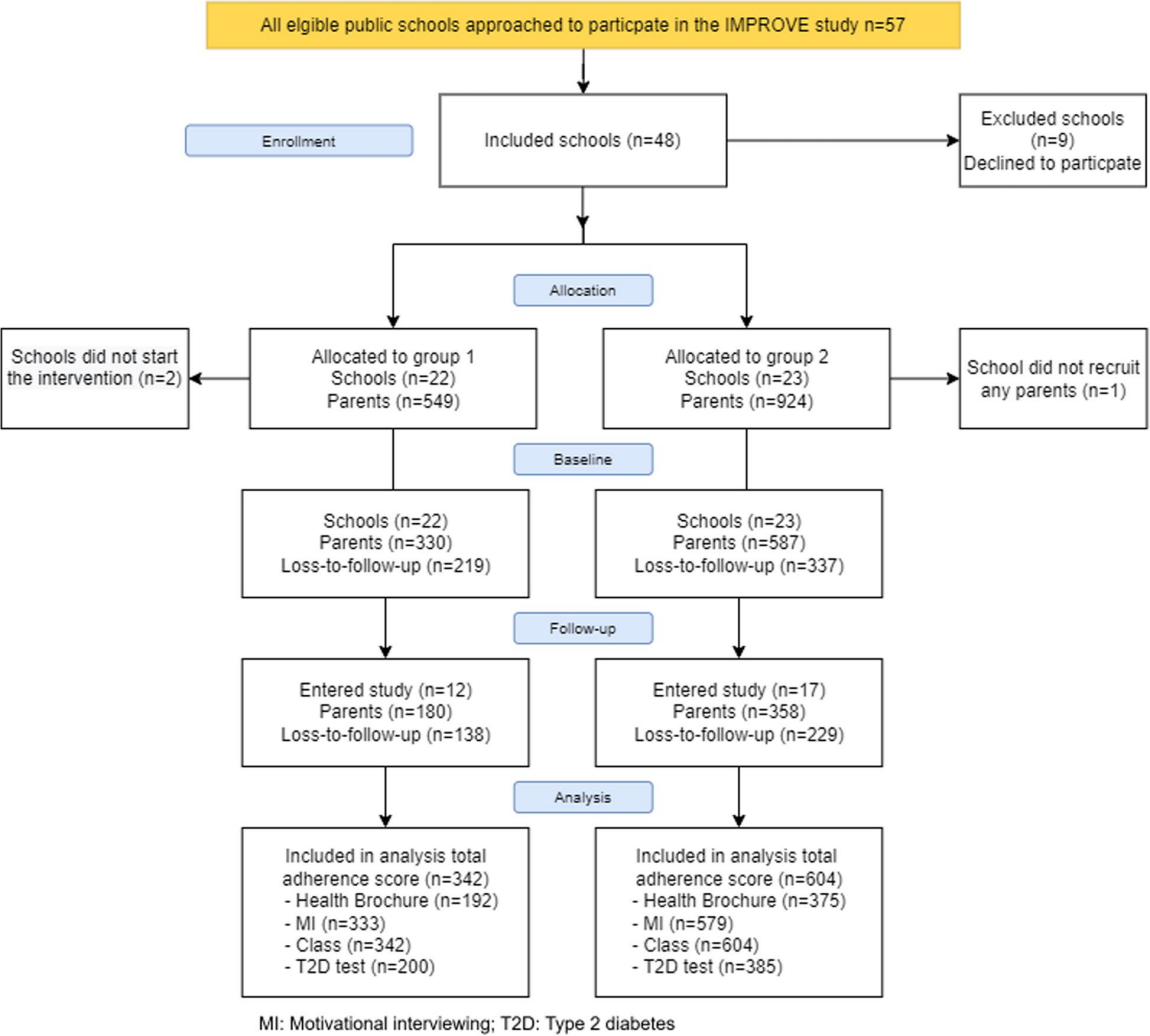
Consort flow-chart of study participants

As illustrated in Fig. 2, a total of 1,474 parents of 794 children from 45 schools consented to participate in the study. Due to organizational changes in the leadership of the school nurses in M2, it was decided by the municipality leadership to end the trial for all schools (*n* = 13) after the first year. In total, 917 parents of 644 children completed the baseline survey. An additional 29 parents and 11 children were enrolled after the baseline data collection period. In total, 567 parents of 383 children completed the follow-up survey.

The majority of parents were women (n = 590, 64.3%) and had post-secondary or university education (642, 70%) (Table 4). Participant characteristics in terms of sex and education were balanced between the groups, however there was a higher proportion of parents born outside of the Nordic countries in the Basic Bundle (group 1) (n = 138, 41.8%) compared to the Enhanced Bundle (group 2) (n = 190, 32.4%). The proportion who filled in the follow-up form did not differ significantly.

**Table 4:** Characteristics of the parents at baseline in the Basic (group 1) and Enhanced Bundle (group 2)

		Baseline	
	Total	Basic Bundle (group 1)	Enhanced Bundle (group 2)
	*n* = 917	*n* = 330	*n* = 587
**Sex**			
Men	327 (35.7%)	114 (34.5%)	213 (36.3%)
Women	590 (64.3%)	216 (65.5%)	374 (63.7%)
**Education**			
Elementary/middle school	64 (7.0%)	16 (4.8%)	48 (8.2%)
High school	211 (23.0%)	83 (25.2%)	128 (21.8%)
Post-secondary education	137 (14.9%)	50 (15.2%)	87 (14.8%)
College/University	505 (55.1%)	181 (54.8%)	324 (55.2%)
**Born in the Nordic countries**			
No	328 (35.8%)	138 (41.8%)	190 (32.4%)
Yes	589 (64.2%)	192 (58.2%)	397 (67.6%)

### Adherence to intervention components

As shown in Table 5, at 12 months, participants in the Enhanced Bundle had a statistically significant higher adherence score compared to those in the Basic Bundle (2.91, SD:1.08 vs 2.70, SD:1.11, *p* = 0.028). Moreover, adherence to the MI health talk component was significantly higher (*p* < 0.001) at 24 months (78.1%) compared to 12 months (65.4%) in the combined sample (Basic + Enhanced). However, in the unadjusted model (Table 6), there was no significant difference between the adherence score (ranging from 0 to 4) in the Basic vs the Enhanced bundle (β = –0.01, *p* = 0.96, 95% CI: –0.31,0.30). Similarly, none of the individual components, reading the health brochure (OR = 1.24, *p* = 0.40, 95% CI: 0.75,2.03), participating in a motivational interviewing (MI) health talk (OR = 0.31, *p* = 0.21, 95% CI: 0.05,1.90), or completing the type 2 diabetes (T2D) risk test (OR = 1.21, *p* = 0.48, 95% CI: 0.72,2.02) were significantly different between participants in the two Bundles of implementation strategies.

**Table 5:** Adherence to intervention components at 12 and 24 months per group (*n* = 946)

	**N**	**12 months**			**24 months**		
**Health Brochure**	946	*Total*	*Group 1 (Basic)*	*Group 2 (Enhanced)*	*Total*	*Group 1 (Basic)*	*Group 2 (Enhanced)*
No		242 (42.7%)	**105 (47.9%)**^**b**^	**137 (39.4%)**^**b**^	169 (44.6%)	58 (44.7%)	114 (44.5%)
Yes		325 (57.3%)	**114 (52.1%)**^**b**^	**211 (60.6%)**^**b**^	210 (55.4%)	68 (55.3%)	142 (55.5%)
**MI Health Talk**	946						
No		**196 (34.6%)**^**a**^	82 (37.4%)	114 (32.8%)	**83 (22.9%)**^**a**^	29 (23.6%)	54 (21.1%)
Yes		**371 (65.4%)**^**a**^	137 (62.6%)	234 (67.2%)	**296 (78.1%)**^**a**^	94 (76.4%)	202 (78.9%)
**Classroom Activities**	946						
Yes		567 (100.0%)	219 (100.0%)	348 (100.0%)	379 (100.0%)	123 (100.0%)	256 (100.0%)
**T2D Risk Test**	946						
No		225 (39.7%)	97 (44.3%)	128 (36.8%)	139 (36.7%)	46 (37.4%)	93 (36.3%)
Yes		342 (60.3%)	122 (55.7%)	220 (63.2%)	240 (63.3%)	77 (62.6%)	163 (63.7%)
**Adherence score [mean, SD]**	946	2.83 (1.10)	**2.70 (1.11)**^***b***^	**2.91 (1.08)**^***b***^	2.97 (1.08)	2.94 (1.12)	2.98 (1.06)

*SD* standard deviation, *MI* Motivational Interviewing, *T2D* Type 2 Diabetes

^*a*^Statistically significant difference between the month 12 and 24

^*b*^Statistically significant difference between Basic (group 1) and Enhanced Bundle (group 2)

**Table 6:** Evaluating the impact of the Basic (group 1) compared to the Enhanced Bundle (group 2) of strategies on the adherence score (model 1) and on individual intervention components (models 2–4)

	Unadjusted Model				Adjusted Model			
Outcome	N	β/OR	*p*-value	95% CI	N	β/OR	*p*-value	95% CI
Model 1: Adherence score (0–4)	946	-.01	0.96	−0.31,0.30	917	0.01	0.95	−0.24,0.25
Model 2: Health brochure (Yes/No)	946	1.24	0.40	0.75,2.03	917	1.18	0.43	0.78,1.79
Model 3: MI Health talk (Yes/No)	946	0.31	0.21	0.05,1.90	917	0.32	0.21	0.06,1.88
Model 4: T2D Risk Test (Yes/No)	946	1.21	0.48	0.72,2.02	917	1.19	0.41	0.78,1.81

Enhanced Bundle (group 2) is compared to Basic Bundle (group 1) (reference). School was a random effect. Adjusted for cohort and parent’s region of birth in all four models

*β* coefficient (model 1), *OR* Odds ratio (models 2–4), *CI* confidence intervals, *MI* Motivational Interviewing, *T2D* Type 2 Diabetes

Results remained consistent in the adjusted model controlling for parents’ region or birth and cohort (12 and 24 months) (Table 6). The adherence score in the Basic bundle continued to show no significant difference compared to the Enhanced implementation strategy bundle (β = 0.01, *p* = 0.95, 95% CI: –0.24,0.25) with a small effect size (Cohen’s d = 0.19).

Likewise, the adjusted odds ratios for receiving the health brochure (OR = 1.18, *p* = 0.43, 95% CI: 0.78,1.79), attending the MI health talk (OR = 0.32, *p* = 0.21, 95% CI: 0.06,1.88), and completing the T2D risk test (OR = 1.19, *p* = 0.41, 95% CI: 0.78,1.81) were not statistically significant between the groups.

In a subgroup analysis, no interaction between the intervention and school size, readiness to implement, parents’ education or region of birth were found. However, when the Basic and Enhanced bundle groups were combined, after adjusting for cohort, parents born within the Nordics had 2.1 higher odds (*p* = 0.006, 95% CI; 1.24,3.46) to complete the MI health talks compared to parents born outside the Nordics.

### Responsiveness to intervention components

On average in both cohorts, the parents’ responsiveness score (i.e., appreciation) was highest for the homework assignments (3.71 SD: 0.90) and lowest for the T2D risk test (3.38: SD: 0.97). The MI health talk and health brochure were scored 3.68 (SD: 0.82) and 3.59 (SD: 0.79) respectively. Table 7 shows the scores between the groups and cohort for each component. When adjusting for school, cohort and parents’ region of birth, the responsiveness to the health brochure (β = –0.06, *p* = 0.41, 95% CI: –0.21,0.08), the MI health talk (β = –0.13, *p* = 0.19, 95% CI: –0.34,0.07), home assignments (β = 0.03, *p* = 0.79, 95% CI: –0.19,0.25) and the T2D risk test (β = –0.08, *p* = 0.50, 95% CI: –0.30,0.14) were not statistically different when compared between the Basic and Enhanced bundle (Table 8).

**Table 7:** Responsiveness of the individual intervention components in the Basic (group 1) versus Enhanced Bundle (group 2)

	n	**12 months**			**24 months**		
		*Total*	*Group 1 (Basic)*	*Group 2 (Enhanced)*	*Total*	*Group 1 (Basic)*	*Group 2 (Enhanced)*
Health Brochure (mean, SD)	535	3.59 (0.76)	3.58 (0.87)	3.60 (0.70)	3.60 (0.83)	3.75 (0.74)	3.52 (0.86)
MI Health Talk (mean, SD)	271	3.75 (0.75)	3.79 (0.53)	3.74 (0.85)	3.56 (0.89)	3.76 (0.91)	3.43 (0.86)
Home assignments (mean, SD)	564	3.70 (0.89)	3.58 (0.97)	3.77 (0.84)	3.72 (0.93)	3.85 (0.97)	3.66 (0.91)
T2D Risk Test (mean, SD)	321	3.37 (0.95)	3.32 (1.00)	3.40 (0.92)	3.40 (1.01)	**3.68 (1.05)**^**a**^	**3.26 (0.97)**^**a**^

The score is from 1–5

*MI* Motivational interviewing, *T2D* Type 2 Diabetes, *SD* Standard deviation aStatistically significant difference between Group 1(Basic) and Group 2 (Enhanced) Bundle

**Table 8:** Evaluating the impact of Enhanced (group 2) versus Basic Bundle (group 1) on the Responsiveness score for each individual intervention component

	Unadjusted Model				Adjusted Model			
Outcome	N	β	*p*-value	95% CI	N	β	*p*-value	95% CI
Model 1: Health Brochure (1–5)	535	−0.08	0.30	−0.22,0.07	509	−0.06	0.41	−0.21,0.08
Model 2: MI Health Talk (1–5)	271	−0.15	0.13	−0.35,0.04	259	−0.13	0.19	−0.34,0.07
Model 3: Home assignments (1–5)	564	0.10	0.39	−0.13,0.33	536	0.03	0.79	−0.19,0.25
Model 4: T2D Risk Test (1–5)	321	−0.12	0.27	−0.35,0.10	313	−0.08	0.50	−0.30,0.14

Enhanced Bundle (group 2) is compared to Basic Bundle (group 1) (reference). School was a random effect. Adjusted for cohort and parent’s region of birth in all four models

*β* Beta coefficient (models 2–4), *CI* confidence intervals, *MI* Motivational Interviewing, *T2D* Type 2 Diabetes

In a sub-analysis combining both groups and adjusting for school and cohort, parents born outside the Nordic countries appreciated the health brochure significantly more (β = 0.21, *p* = 0.008, 95% CI: 0.06,0.37) and also gave higher scores to the T2D risk test (β = 0.53, *p* < 0.001, 95% CI: 0.27,0.78) compared to those born in the Nordic countries.

### Fidelity to implementation strategies

Most of the Basic Bundle implementation strategies were performed as planned. During the checklist meetings at the end of the first school year, it became clear that implementation strategy #5 (*change/alter environment*) in the Basic Bundle was interpreted differently among the schools and had not been performed for various reasons. It varied from rebuilding the school yard to minor changes in the school meals. Many schools reported there was no need to change/alter their environment at all. We therefore decided to exclude this strategy from the evaluation.

**Fig. 3:**
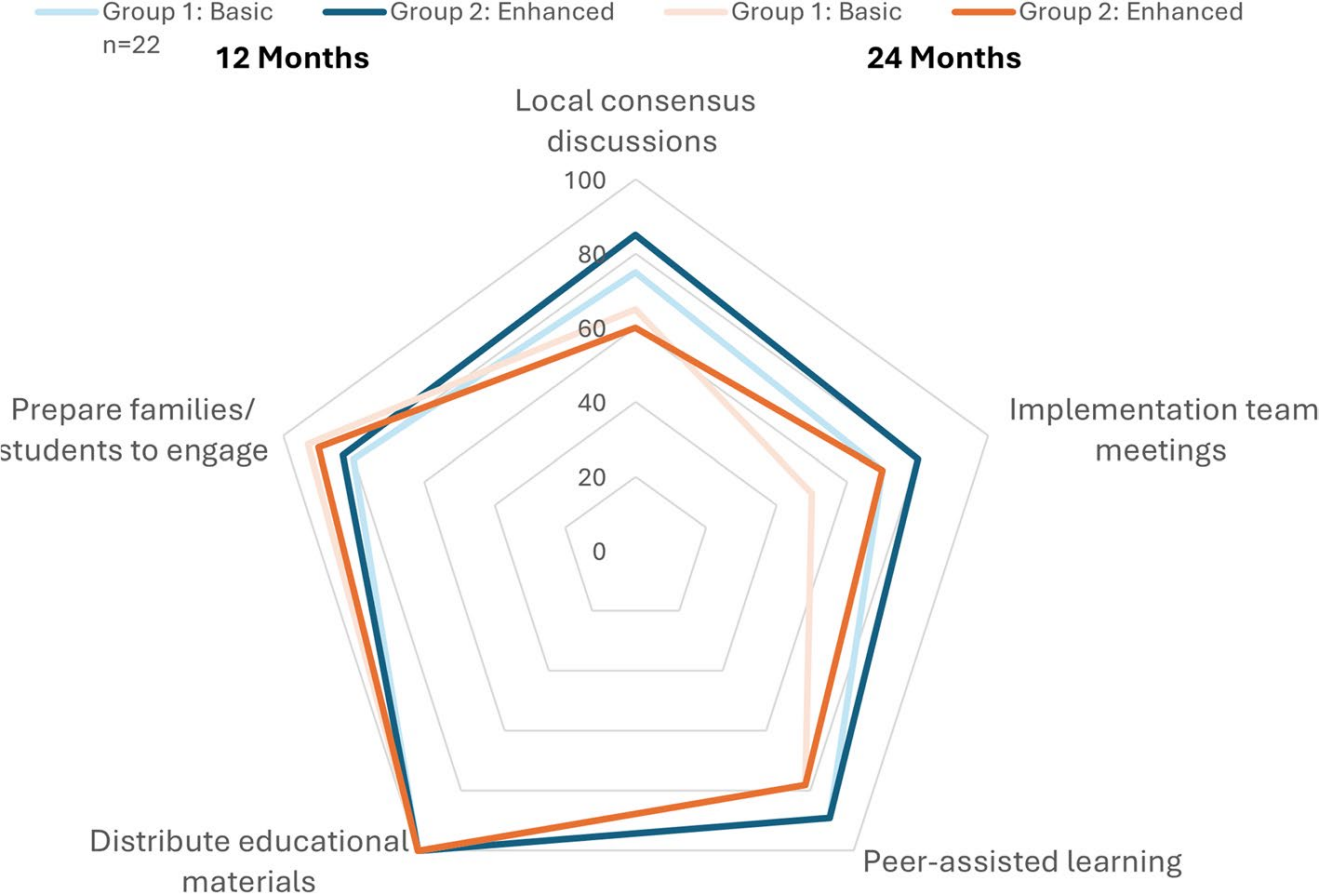
Proportion completed of Basic Bundle (group 1) implementation strategies (*n* = 9) at 12 and 24 months

The average score for the Basic Bundle (five strategies) was 14.8 (78%) out of 18 points. No statistically significant difference was detected between the schools assigned to the Basic Bundle (14.5 [76%]) and the Enhanced Bundle (15.1 [79%]) for the five Basic Bundle strategies. However, the total average score for all schools at month 12 (15.7 [87%]) was significantly higher (*p*-value = 0.002) than at month 24 (13.5 [75%]). All schools *distributed the educational material* as designed at both times. *Preparing families to be active participants* increased over time, however the other three strategies decreased (Fig. 3) as school personnel became more comfortable with running the new program.

Among Enhanced Bundle-schools, only the strategy *provide ongoing consultation/coaching* was implemented at 12 months. *Educational outreach visits* were offered, however no school requested these sessions. While a yearly information letter was sent to the primary health care centers (PHCs) in each municipality about the HSS program and the IMPROVE study from the research team to facilitate the *promote network weaving strategy*, no additional activities were performed between the PHCs and the schools. Some school nurses reported that communication with the PHCs functioned well, while others felt the referral process for families who needed additional support (not part of this study) was not optimal. The *coaching* was planned to take place in an online forum due to the ongoing Covid-19 pandemic but this was changed to a one-on-one online coaching meeting with each school after four months due to low participation in the forum. Finally, the *feedback to school personnel* regarding responsiveness of parents (strategy 10) was only given during the second year because data was not yet available during the first year.

The average additional score for the schools receiving the Enhanced bundle at 12 months was 1.3 (26%) out of 5 points possible. This improved significantly (*p* < 0.001) to 2.7 points (54%) at 24 months. This was driven by all schools *obtained and used student and family feedback* and the *consultation*/*coaching* increased to 1.7 points with 85% of the schools having the recommended two coaching sessions compared to 1.3 points (65%) at 12 months.

## Discussion

The findings of this study indicate that overall fidelity to the HSS program was around 75%, with most components implemented as expected. However, additional tailored implementation strategies in the Enhanced bundle, offering more external support, did not lead to significantly better fidelity to the HSS intervention components compared to the Basic bundle, as hypothesized. These results indicate that school staff have the capability to implement HSS with relatively high fidelity after receiving 1–2 h of online training, a two-day MI training course for school nurses and the basic implementation strategy bundle. This consisted of consensus building, formation of an implementation team to coordinate the program locally, knowledge exchange between school personnel, access to implementation manuals and motivating parents to participate.

While not significant, the adherence score in the Enhanced group showed a tendency to be higher at 12 months. This difference was not seen at 24 months indicating that the schools were able to achieve an equal level of adherence after two years of implementing the program. The improvements seen were driven by an increased adherence to the MI health talk component in the group receiving the Basic bundle. This suggests that the school nurses conducted more talks and found it easier to implement overtime, as also noted in previous studies [30]. It is well documented in the literature that adapting to new routines requires a prolonged period for stabilization and integration into practice. Similarly in our study, MI takes time to learn and becomes easier to integrate into daily routines with increasing practice. In qualitative interviews conducted after the study's conclusion, school personnel reported a perceived initial steep learning curve and significant effort required to implement the HSS program noting that staff became more comfortable with the process after the first year [31].

Several reasons could explain why we saw no difference in fidelity (adherence or responsiveness to the intervention components), after adjusting for covariates, between the two bundles of implementation strategies. The most likely reason is that the Basic Bundle in conjunction with the pre-implementation strategies based on experience from previous studies was comprehensive and led to high adherence. Secondly, the yearly study-related checklist meeting with all schools served as an unintentional but probably effective audit and feedback session. Thirdly, two of the four strategies in the Enhanced Bundle of implementation strategies were not used. When asked directly, the schools did not feel a need for *educational outreach visits* contrary to what was discussed in the workshops with stakeholders during the design phase of the study [19]. Regarding primary health care, although initially positive to the HSS program and the strategy itself, they could not prioritize time for networking with schools.

A recent Cochrane review evaluated strategies to improve school implementation of interventions targeting students' diet, physical activity, obesity, tobacco use and/or alcohol use [15]. The most common strategies were educational materials, meetings, outreach visits, and local consensus processes, similar to our study. The review found that using an implementation strategy bundle likely resulted in higher fidelity in randomized controlled trials compared to no active implementation strategy. However, when analyzing studies similar to IMPROVE that also compare two or three different bundles of strategies (i.e., hybrid type III studies), no or small positive effects were found. This may indicate that well-designed bundles of implementation strategies are needed up to a certain level after which it becomes difficult to achieve additional benefits.

The implementation strategies used in our study are commonly applied in other studies. A systematic review of experimentally tested implementation strategies across health and human service settings found that educational strategies were the most frequently used, as they are considered necessary but not sufficient on their own. In contrast, pre-implementation strategies showed strong evidence of effectiveness [32]. As described in the methods, we also used several pre-implementation strategies [19]. In addition, an implementation manual, regarded as a highly important and feasible implementation strategy [33] was also available for all schools (included in the Basic strategy *Distribute educational materials*), which probably contributed to leveling out the impact of the two implementation strategy bundles.

In our study, parental responsiveness scores did not differ between schools implementing the Basic and Enhanced Bundles, likely because school personnel in both groups received identical training, resulting in comparable competence prior to the intervention. However, specific intervention components resonated differently with parents; homework assignments were the most engaging, while the type 2 diabetes (T2D) risk test was the least. Notably, parents born outside the Nordic countries, which another study found tend to have a higher prevalence and earlier onset of T2D [34] compared to Swedish-born inhabitants, demonstrated higher responsiveness to the T2D risk test. This could suggest a greater perceived relevance of the test within this group.

Moreover, parents born in Nordic countries had twice the odds to complete the MI health talks. This indicates that the cultural background of parents may significantly influence adherence to this component. This is an important finding since a long-term follow up of the HSS program showed that children from families with high fidelity to the intervention, especially the MI health talk had better long-term diet outcomes compared to controls [35]. Thus, to enhance engagement among non-Nordic parents and address the needs of diverse parent groups, more tailoring of the strategy *Prepare families and students to be active participants* will be needed.

Regarding fidelity to the implementation strategies, we saw a slight decrease over time for all strategies except for the strategy *distribute educational materials* and *Prepare families and students to be active participants*. The latter actually increased which could be explained by that the school personnel as well as the research team realized and discussed the importance of engaging all parents during the yearly checklist meeting for all schools.

### Methodological considerations

In the context of school-based research, there are limited examples of hybrid type-3 studies with large sample sizes. A strength of this study is its cluster-randomized design, which allows for the assessment of the comparative effectiveness of bundles of implementation strategies on program fidelity. Understanding fidelity from the perspectives of school personnel (deliverers) and parents (recipients) was important for the study. However, this understanding was somewhat compromised due to relatively low response rate by parents regarding the health brochure and T2D test, which contributed to a weakening of the fidelity measures. This may have yielded a lower adherence score to these two components than in reality.

The parents in our study appeared to have a higher level of education compared to the average in their municipality, suggesting a selection bias that may limit the generalizability of our findings. Implementing interventions is often more feasible with families that have more resources (e.g., higher income or education), as they may have greater access to information, support networks, and the capacity to engage with new programs. Dropout and selection biases should be considered, as they can affect the representativeness of the sample and the applicability of the results to broader populations.

The trial began in 2021 during the COVID-19 pandemic, when primary schools in Sweden remained open and teaching continued as usual. However, due to restrictions on in-person meetings, there was a necessary shift to online meetings. This included training of staff and coaching sessions. Some of the school nurses reported that the motivational interviews took place online. This also had some advantages as it allowed for increased flexibility in the training and coaching approaches. By following the program for two years, we could see an improvement in the MI health talk underlining that a certain degree of maturity occurs with program repetition.

The research team assessed fidelity to the implementation strategies using a checklist during school visits (“Monitor the progress of implementation effort”). The school visits unintentionally provided feedback on implementation performance to all schools, which was not intended originally. So while evaluating fidelity through direct observation was a strength, an alternative approach could have been a survey to limit the unintentional feedback to the schools only receiving the Basic Bundle.

### Main learnings from the study

Involvement of school personnel was essential to define and tailor the implementation strategies. However, reflecting on our approach, it is evident that including parents in the study design process would have been advantageous, especially in the tailoring of the strategy “*Prepare families and students to be active participants*”. This is particularly true for non-Nordic parents with a different cultural background, who had a lower adherence to the MI health talk.

It can be questioned if the hybrid type 3 design is optimal considering the complexity and duration of the program and that the implementation strategies were not pre-tested. As an alternative to a randomized control trial, an adaptive iterative implementation trial would have allowed for continuous improvement, to ensure feasibility and acceptability of all implementation strategies. Adaptive iterative designs are ideal when there is uncertainty about which implementation strategies to use and when the sequence or combination of implementation strategies needs to be pilot tested [36].

Our findings also raise broader implications for implementation research methodology. Although the Enhanced bundle of strategies was designed based on barriers and facilitators such as providers’ competence and attitudes towards the program and lack of collaboration between primary healthcare and school this did not lead to the expected improvements in intervention fidelity. In hindsight, these determinants may not have been the most important ones. In our study, 28 determinants were identified, but their relative importance was not established. While the CFIR provides a comprehensive way of identifying determinants at multiple levels, it offers little guidance on how to prioritize among barriers or facilitators, or on which are most amenable to change. This is probably also very contextually determined and difficult to generalize. Second, although SISTER and similar taxonomies provide a structured set of strategies, it is not always clear beforehand whether the selected strategies are feasible in practice to meaningfully address the determinants they target. Our finding that fidelity varied more by parents’ country of birth than by study arm illustrates this challenge.

These limitations suggest that null findings in implementation trials may reflect not only sample- or study-specific factors, but also the current limits of our methodological tools for linking determinant identification and prioritization to effective strategy development. The challenges of selecting and tailoring strategies to address identified barriers have been discussed for nearly a decade, with several papers emphasizing the importance of contextual sensitivity [37]. Building on this work, we propose an additional step in the process: ranking identified barriers prior to strategy selection and tailoring. In doing so, our study contributes to the growing body of research that seeks to advance the methodological toolkit of implementation science by emphasizing the value of systematically prioritizing contextual barriers before matching them with implementation strategies.

## Conclusions

This study contributes to the growing field of literature on the effectiveness of tailored implementation strategies when scaling up a research-based intervention to real-world practice. The main outcome, fidelity to the four intervention components assessed as adherence, was relatively high. However, no difference in adherence to the intervention was found between the Basic and Enhanced implementation strategy bundles at 12 and 24 months. This could most likely be explained by the extensive pre-implementation strategies employed by the research team, a comprehensive Basic bundle, and a partly ineffective Enhanced bundle of implementation strategies. We therefore conclude that relatively high fidelity to all components of the HSS program can be achieved through a combination of: (1) Introductory training for school personnel and access to a centralized online technical assistance platform with a manual and educational materials provided by an external actor; (2) implementation of the basic bundle of strategies by school staff, complemented by an end-of-year audit and feedback meeting facilitated by an external actor; and (3) additional targeted measures to support and engage foreign-born parents born as active participants in the program.

## Supplementary Information

Additional file 1. (CONSORT Cluster) Statement.

Additional file 2. Standards for Reporting Implementation Studies (StaRI) guidelines.

Additional file 3. Major deviations to the study protocol and rationale for deviations.

Additional file 4. Implementation strategy checklist.

## Data Availability

Data will be available upon request from the corresponding author.

## Abbreviations

BMI: Body Mass Index
CFIR: Consolidated Framework for Implementation Research
FINDRISC: Finnish Diabetes Risk Score
HSS: Healthy School Start
IMPROVE: IMplementation and evaluation of the school-based family support PRogram a Healthy School Start to promote child health and prevent OVErweight and obesity
MI: Motivational Interviewing
M1,2,3: Municipality
N: Nurse
P: Parent
REDCap: Research Electronic Data Capture
T: Teacher
T2D: Type 2 diabetes
