# Supplementary material for: Comparing tailored implementation strategies to improve intervention fidelity in a school-based obesity prevention program: the IMPROVE hybrid type III trial
Source: Implement Sci. 2025 Dec 28;21:17. doi: 10.1186/s13012-025-01481-0 (PMC12924443; doi:10.1186/s13012-025-01481-0)
Supplement: Supplementary file 1 — Additional file 1. (CONSORT Cluster) Statement. [file 13012_2025_1481_MOESM1_ESM.pdf]

CONSORT 2010 checklist of information to include when reporting a cluster randomised trial*

Section/Topic	Item No	Checklist item	Reported on page No
**Title and abstract**			
	1a	Identification as a cluster randomised trial in the title	1
	1b	Structured summary of trial design, methods, results, and conclusions (for specific guidance see CONSORT for abstracts)	1
**Introduction**			
Background and objectives	2a	Scientific background and explanation of rationale, *including the rationale for using a cluster design*	2-3
	2b	Specific objectives or hypotheses. Whether objectives pertain to the cluster level, the individual participant level, or both	3
**Methods**			
Trial design	3a	Description of trial design (such as parallel, factorial) including allocation ratio. Definition of cluster and description of how the design features apply to the clusters	3
	3b	Important changes to methods after trial commencement (such as eligibility criteria), with reasons	Additional file 3
Participants	4a	Eligibility criteria for clusters	3-4
	4b	Settings and locations where the data were collected	3-4
Interventions	5	Precise details of the interventions intended for each group, *whether they pertain to the individual level, the cluster level, or both*, and how and when they were actually administered	4-7
Outcomes	6a	Clearly defined primary and secondary outcome measures, *whether they pertain to the individual level, the cluster level, or both*, and, when applicable, any methods used to enhance the quality of measurements (eg multiple observations, training of assessors)	7-8
	6b	Any changes to trial outcomes after the trial commenced, with reasons	8-9
Sample size	7a	How *total* sample size was determined *(including method of calculation, number of clusters, cluster size, a coefficient of intracluster correlation (ICC or k), and an indication of its uncertainty)* and, when applicable, explanation of any interim analyses and stopping rules	8
	7b	When applicable, explanation of any interim analyses and stopping guidelines	N/A
Randomisation:			
Sequence generation	8a	Method used to generate the random allocation sequence	3
	8b	Type of randomisation; details of any restriction (such as blocking, block size, stratification, matching).	8-9
Allocation	9	Mechanism used to implement the random allocation sequence, *specifying that allocation was based on*	8-9

concealment mechanism		*clusters rather than individuals and clarifying* whether the sequence was concealed until interventions were assigned	
Implementation	10a	Who generated the random allocation sequence, who enrolled clusters, and who assigned clusters to interventions	3
	10b	Mechanism by which individual participants were included in clusters for the purposes of the trial (such as complete enumeration, random sampling)	
	10c	From whom consent was sought (representatives of the cluster, or individual cluster members, or both) and whether consent was sought before or after randomisation	4
Blinding	11a	If done, who was blinded after assignment to interventions (for example, participants, care providers, those assessing outcomes) and how	4
	11b	If relevant, description of the similarity of interventions	N/A
Statistical methods	12a	Statistical methods used to compare groups for primary and secondary outcomes indicating how clustering was taken into account	8-10
	12b	Methods for additional analyses, such as subgroup analyses and adjusted analyses	9-10
**Results**			
Participant flow (a diagram is strongly recommended)	13a	For each group, the numbers of clusters that were randomly assigned, received intended treatment, and were analysed for the primary outcome	8-10
	13b	For each group, losses and exclusions for both clusters and individual cluster members, together with reasons	9
Recruitment	14a	Dates defining the periods of recruitment and follow-up	3-4
	14b	Why the trial ended or was stopped	N/A
Baseline data	15	A table showing baseline information for each group for the individual and cluster levels as applicable	10
Numbers analysed	16	Number of *clusters and* participants (denominator) in each group included in each analysis and whether the analysis was by intention to treat. State the results in absolute numbers when feasible (e.g., 10/20 not 50%)	8-10
Outcomes and estimation	17a	For each primary and secondary outcome, a summary of results for each group for the individual or cluster level as applicable, and the estimated effect size and its precision (eg 95% confidence interval) and a coefficient of intracluster correlation (ICC or k) for each primary outcome.	10-13
	17b	For binary outcomes, presentation of both absolute and relative effect sizes is recommended	
Ancillary analyses	18	Results of any other analyses performed, including subgroup analyses and adjusted analyses, distinguishing pre-specified from exploratory	12-13
Harms	19	All important harms or unintended effects in each group (for specific guidance see CONSORT for harms)	N/A
**Discussion**			
Limitations	20	Trial limitations, addressing sources of potential bias, imprecision, and, if relevant, multiplicity of analyses	14-15
Generalisability	21	Generalisability to clusters and/or individual participants (as relevant)	14-15

Interpretation	22	Interpretation consistent with results, balancing benefits and harms, and considering other relevant evidence	N/A
**Other information**			
Registration	23	Registration number and name of trial registry	1
Protocol	24	Where the full trial protocol can be accessed, if available	1
Funding	25	Sources of funding and other support (such as supply of drugs), role of funders	16

Citation: Schulz KF, Altman DG, Moher D, for the CONSORT Group. CONSORT 2010 Statement: updated guidelines for reporting parallel group randomised trials. BMC Medicine. 2010;8:18.
© 2010 Schulz et al. This is an Open Access article distributed under the terms of the Creative Commons Attribution License (http://creativecommons.org/licenses/by/2.0), which permits unrestricted use, distribution, and reproduction in any medium, provided the original work is properly cited.

*We strongly recommend reading this statement in conjunction with the CONSORT 2010 Explanation and Elaboration for important clarifications on all the items. If relevant, we also recommend reading CONSORT extensions for cluster randomised trials, non-inferiority and equivalence trials, non-pharmacological treatments, herbal interventions, and pragmatic trials. Additional extensions are forthcoming: for those and for up-to-date references relevant to this checklist, see www.consort-statement.org.
