# Supplementary material for: Comparing tailored implementation strategies to improve intervention fidelity in a school-based obesity prevention program: the IMPROVE hybrid type III trial
Source: Implement Sci. 2025 Dec 28;21:17. doi: 10.1186/s13012-025-01481-0 (PMC12924443; doi:10.1186/s13012-025-01481-0)
Supplement: Supplementary file 3 — Additional file 3. Major deviations to the study protocol and rationale for deviations. [file 13012_2025_1481_MOESM3_ESM.pdf]

Additional file 3. Major deviations to the study protocol and rationale for deviations

**Topic**	**Protocol**	**Deviation**	**Rationale**
Cohort	All children starting pre-school class (5–7 years of age) during the school year 2021/22 and 2022/23 and their parents/guardians are eligible for the study.	Added a 3rd municipality (M) and an additional school year (23/24)	M3 was interested in participating in the study, however their participation was delayed one year due to the Covid-19 pandemic
Fidelity measurements: Quality of delivery	Quality of Motivational Interviewing (MI) score coded according to MITI 4.2	Assessment of quality of delivery was not conducted	It was decided to discontinue the recording of the MI sessions as it was perceived as a significant deterrent for the nurses to conduct the MI
Fidelity measurements: Adherence for the T2D test	Calculate the proportion of parents with high-risk scores that subsequently attend health care	The proportion of parents with high-risk scores was not available.	We were not able to engage the primary health care centers in the referral process and this data was not collected from the parents.
Fidelity to implementation strategies assessment	Each strategy will be graded as 0 = not implemented, 1 = partially implemented, 2 = fully implemented.	Each strategy was graded as completed (1) or not (0)	During piloting, the three-level grading system proved difficult to apply consistently, particularly in distinguishing partial from full implementation. To enhance clarity and inter-rater reliability, the fidelity assessment was modified to a binary scoring of completed (1) versus not completed (0).
Organizational readiness to implement	There are three components to the score: 1) A total readiness score; 2) a change efficacy score 3) and a change commitment score.	One component	A validation study (under review) was conducted and found only one construct in the Swedish setting
