# Supplementary material for: Comparing tailored implementation strategies to improve intervention fidelity in a school-based obesity prevention program: the IMPROVE hybrid type III trial
Source: Implement Sci. 2025 Dec 28;21:17. doi: 10.1186/s13012-025-01481-0 (PMC12924443; doi:10.1186/s13012-025-01481-0)
Supplement: Supplementary file 4 — Additional file 4. Implementation strategy checklist. [file 13012_2025_1481_MOESM4_ESM.pdf]

Checklist to monitor fidelity to implementation strategies

- In the visit done to each school (Group 1 and Group 2) the blue items on the checklist will be documented.
- Group 2 schools (Enhanced) will also be asked the yellow items on the checklist.
- Feedback about the implementation will be given later.

School name: **Who attended**: **Date:** **Group**:

**Targeted strategy** (SISTER taxonomy number)	**Respondent**	**Question**	**Scoring Criteria – Yes (1) or No (0)**	**Comment**
1. Conduct local consensus discussions – Reach common view about the HSS (23)	School principal	1.1 Introduced the HSS to all school personnel to reach consensus		
		1.2 Participated in a planning meeting for the implementation of the HSS in the school		
2. Distribute educational materials (42)	HSS-team coordinator in each school	Ordered and distributed written material at the beginning of the new school year		
3. Organize an HSS-team in the school (32)	School principal	3.1 Appointed a coordinator for the HSS-team<br><br>Who?		
	HSS-team coordinator in each school	3.2 Created an HSS-team OR included a teacher representative in the school health team to implement the HSS		
		3.3 Held more than one meeting with the HSS-team to discuss how the practical work will be distributed, the implementation process, how to support each other and exchange knowledge		

4. Peer-assisted learning and knowledge exchange among school staff involved in HSS (13)	Teachers in each school	4.1 Read the teacher's manual		
		4.2 Watched the introductory video to the classroom component		
		4.3 Made a plan for implementation and how to communicate with parents about the home assignments		
		4.4 Assist each other regarding organisation of work with HSS program		
	School nurse	4.5 Did the MI-training		
		4.6 Received feedback via phone after the MI-training		
		4.7 Read the manual		
		4.8 Read the information about HSS on the website		
	Principal	4.9 Read the information about HSS on the website and in the manual		
5. Change / alter environment (54)	HSS-team coordinator in each school	5.1 Discussed the possibility to make changes within school and around the school to support the new program		
6. Prepare and motivate families to be active participants (55)	School principal	6.1 Declared to caregivers that the schools is a health promoting school		
	HSS-team coordinator in each school	6.2 Introduced the HSS at the first meeting with parents (showed introductory film to caregivers or sent the link to the film)		
	Teachers in each school	6.3 Information sent out to parents again when the classroom component started		

7. Promote network weaving with primary health care (33)	HSS-team coordinator in each school	7.1 Held yearly meetings with the primary health care for information sharing and collaborative problem-solving and shared goals regarding family health		
8. Provide ongoing consultation/coaching (44)	HSS-team	8.1 Discussed the feedback report on implementation strategies from the RT and how to improve implementation		
		8.2 Communicated with the school personnel about how caregivers perceive the program		

Abbreviations: HSS = Healthy School Start

Additional questions

- Have there been any major changes at the school during the past school year which could have affected the program or the study?
- How can we increase the number of caregivers that consent to participate in the IMPROVE study?
- Have all caregivers in the study received health talks with MI? If not, which ones?
- How have you followed up families that need more support (those with overweight or unhealthy habits)?
- If not all pre-school or year 1 classes participate in the program, why?
- Do you have ideas on how we can continue the preventative work with older children in the school? Specially those in Year 4.
- What is your opinion on the collaboration with the primary health care unit in your area in relation to family support for health promotion? Do you have contact regarding those issues?
